# Ectopic and Visceral Fat Deposition in Lean and Obese Patients With Type 2 Diabetes

**DOI:** 10.1016/j.jacc.2016.03.597

**Published:** 2016-07-05

**Authors:** Eylem Levelt, Michael Pavlides, Rajarshi Banerjee, Masliza Mahmod, Catherine Kelly, Joanna Sellwood, Rina Ariga, Sheena Thomas, Jane Francis, Christopher Rodgers, William Clarke, Nikant Sabharwal, Charalambos Antoniades, Jurgen Schneider, Matthew Robson, Kieran Clarke, Theodoros Karamitsos, Oliver Rider, Stefan Neubauer

**Affiliations:** a University of Oxford Centre for Clinical Magnetic Resonance Research, Radcliffe Department of Medicine, Division of Cardiovascular Medicine, Oxford, United Kingdom; b Department of Physiology, Anatomy and Genetics, University of Oxford, Oxford, United Kingdom; c Translational Gastroenterology Unit, University of Oxford, Oxford, United Kingdom; d Perspectum Diagnostics Ltd., Oxford, United Kingdom; e Division of Cardiovascular Medicine, University of Oxford, Oxford, United Kingdom; f Cardiology Department, Oxford University Hospitals NHS Foundation Trust, Oxford, United Kingdom

**Keywords:** diabetic cardiomyopathy, epicardial fat deposition, fatty liver disease, magnetic resonance imaging, magnetic resonance spectroscopy, ^1^H-MRS, proton magnetic resonance spectroscopy, ^31^P-MRS, phosphorus magnetic resonance spectroscopy, ATP, adenosine triphosphate, BMI, body mass index, BP, blood pressure, CT, computed tomography, cT_1_, iron-corrected T_1_, HOMA-IR, homeostasis model assessment of insulin resistance, Ln-T2D, lean patients with type 2 diabetes, LV, left ventricular, MR, magnetic resonance, MRI, magnetic resonance imaging, NAFLD, nonalcoholic fatty liver disease, Ob-T2D, obese patients with type 2 diabetes, PCr, phosphocreatine, T2D, type 2 diabetes

## Abstract

**Background:**

Type 2 diabetes (T2D) and obesity are associated with nonalcoholic fatty liver disease, cardiomyopathy, and cardiovascular mortality. Both show stronger links between ectopic and visceral fat deposition, and an increased cardiometabolic risk compared with subcutaneous fat.

**Objectives:**

This study investigated whether lean patients (Ln) with T2D exhibit increased ectopic and visceral fat deposition and whether these are linked to cardiac and hepatic changes.

**Methods:**

Twenty-seven obese patients (Ob) with T2D, 15 Ln-T2D, and 12 normal-weight control subjects were studied. Subjects underwent cardiac computed tomography, cardiac magnetic resonance imaging (MRI), proton and phosphorus MR spectroscopy, and multiparametric liver MR, including hepatic proton MRS, T_1_- and T_2_*-mapping yielding “iron-corrected T_1_” [cT_1_].

**Results:**

Diabetes, with or without obesity, was associated with increased myocardial triglyceride content (p = 0.01), increased hepatic triglyceride content (p = 0.04), and impaired myocardial energetics (p = 0.04). Although cardiac structural changes, steatosis, and energetics were similar between the T2D groups, epicardial fat (p = 0.04), hepatic triglyceride (p = 0.01), and insulin resistance (p = 0.03) were higher in Ob-T2D. Epicardial fat, hepatic triglyceride, and insulin resistance correlated negatively with systolic strain and diastolic strain rates, which were only significantly impaired in Ob-T2D (p < 0.001 and p = 0.006, respectively). Fibroinflammatory liver disease (elevated cT_1_) was only evident in Ob-T2D patients. cT_1_ correlated with hepatic and epicardial fat (p < 0.001 and p = 0.01, respectively).

**Conclusions:**

Irrespective of body mass index, diabetes is related to significant abnormalities in cardiac structure, energetics, and cardiac and hepatic steatosis. Obese patients with T2D show a greater propensity for ectopic and visceral fat deposition.

Type 2 diabetes (T2D) and obesity are both associated with nonalcoholic fatty liver disease (NAFLD), cardiomyopathy 1, 2, and increased cardiovascular mortality 3, 4. The incidence of T2D continues to increase, driven predominantly by the obesity epidemic. Although obesity is likely to be a strong contributor to diabetic cardiomyopathy (5), many patients with diabetic cardiomyopathy have normal body mass index (BMI), suggesting that diabetes and obesity may have different mechanisms by which they mediate cardiovascular change and that diabetic cardiomyopathy may occur in patients with T2D without obesity. Furthermore, evidence suggests that distribution of excess fat is an important determinant of cardiovascular risk, and ectopic and visceral adiposity confer a much higher risk than subcutaneous adiposity 6, 7.

Ectopic and visceral fat storage may be linked to insulin resistance, and it is widely known that insulin resistance is the strongest predictor of development of diabetes (8). Increasing evidence points to a strong association between insulin resistance and nonischemic heart failure (9), although there are differing opinions regarding whether this relationship is of a protective or pathological nature 10, 11, 12. Thus, the presence of ectopic and visceral fat deposition in patients with T2D even in the absence of a global increase in total body fat may potentially play a significant role in this association. Assessing body composition is, therefore, likely to be more important in patients with T2D than simple metrics of obesity. Liver fat is considered a key feature of ectopic fat associated with dysfunctional adipose tissue and visceral fat deposition (13), and there is also growing interest in the imaging of epicardial adipose tissue as a proxy measure of visceral fat.

Epicardial adipose tissue, a form of visceral fat, may affect the underlying myocardium by secreting a wide range of adipokines (14). Furthermore, excess liver fat has been shown to be accompanied by cardiac structural and functional changes (15). Computed tomography (CT) allows quantification of epicardial fat volume, and proton magnetic resonance spectroscopy (^1^H-MRS) allows quantification of lipid content in the liver and the heart. Multiparametric magnetic resonance (MR) of the liver, including ^1^H-MRS for assessment of steatosis and T_1_ and T_2_* mapping (yielding iron corrected T_1_ [cT_1_]) (16), allows noninvasive quantification of liver fat and identification of the presence of hepatic fibroinflammatory disease with a high diagnostic accuracy (16).

Myocardial energetic compromise is an important feature of both the diabetic (17) and the nondiabetic obese heart (5). However, changes in cardiac energy metabolism in lean patients with diabetes have not been previously studied. Myocardial phosphocreatine to adenosine triphosphate concentration ratio (PCr/ATP) is a sensitive indicator of the myocardial energy status, and phosphorus magnetic resonance spectroscopy (^31^P-MRS) allows noninvasive assessment of the PCr/ATP.

Our primary aim was to test the hypothesis that lean patients (Ln) with T2D exhibit increased ectopic and visceral fat deposition. Our secondary aim was to test whether or not ectopic and visceral adiposity in diabetes is associated with insulin resistance and cardiac and hepatic changes. We used cardiac CT, multiparametric liver magnetic resonance imaging (MRI), cardiac MRI, ^1^H-MRS, and ^31^P-MRS to assess and compare epicardial, hepatic, and myocardial fat deposition; hepatic fibroinflammatory changes; and cardiac structure, function, and energetics in lean and obese patients (Ob) with T2D and in control subjects without diabetes.

## Methods

The study was approved by the National Research Ethics Committee (Ref 13/SW/0257), and informed written consent was obtained from each participant. Patients were recruited from general practice surgeries in Oxfordshire, United Kingdom. A total of 27 Ob-T2D, 15 Ln-T2D, and 12 healthy normal weight control subjects were recruited to the study. We have previously reported changes in myocardial energetics, triglyceride content, and left ventricular (LV) structure and function in patients with diabetes compared with healthy volunteers 18, 19. Using this database, and expanding the data with novel recruitment of 12 healthy volunteers to the study, here we report a comparison of the changes in these cardiac features in 2 subgroups of patients with diabetes (obese and lean) compared with healthy volunteers. Additionally, we report an analysis of epicardial fat volumes, liver triglyceride content, and liver fibroinflammatory changes.

### Exclusion criteria

Subjects were excluded if they had a previous diagnosis of cardiovascular or liver disease, hypertension (resting systolic blood pressure >140 mm Hg and diastolic blood pressure >90 mm Hg), contraindications to MRI, ischemic changes on 12-lead electrocardiography, or renal impairment (estimated glomerular filtration rate <30 ml/min/1.73 m^2^); if they were tobacco smokers; if their alcohol intake was above 21 units in a week for men or 14 units for women; or if they were insulin dependent.

**Figure 1:**
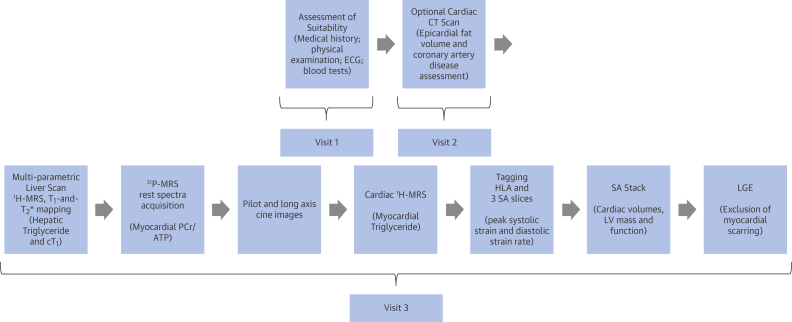
Study Protocol for Patients With T2D Suitability of patients with type 2 diabetes (T2D) was assessed during the first hospital visit. Those patients who consented to have a cardiac computed tomography (CT) scan were then invited for the second hospital visit. The third hospital visit included magnetic resonance imaging (MRI) and magnetic resonance spectroscopy scans (3T). Multiparametric liver MRI included proton magnetic resonance spectroscopy (^1^H-MRS) for hepatic triglyceride; T_1_ and T_2_* mapping yielded iron-corrected T_1_ (cT_1_). This was followed by cardiac magnetic resonance, which included cine imaging to assess left ventricular (LV) volumes, mass, and ejection fraction; myocardial tagging for assessment of peak circumferential systolic strain and diastolic strain rate; cardiac ^1^H-MRS for myocardial triglyceride; and late gadolinium enhancement (LGE) imaging for exclusion of myocardial scarring. Control subjects underwent identical MRI protocols. ^31^P-MRS = phosphorus magnetic resonance spectroscopy; ECG = electrocardiogram; HLA = horizontal long axis; PCr/ATP = myocardial phosphocreatine to adenosine triphosphate concentration ratio; SA = short axis.

Control subjects had no history of heart disease, diabetes mellitus (fasting glucose level ≥6.7 mmol), or hypertension and were not taking any medications. Study assessments were carried out on a single visit for the healthy control subjects and over 2 or 3 visits for patients with T2D, depending on individuals’ consent for attending cardiac CT assessments (Figure 1).

### Anthropometric measurements

Height and weight were recorded and BMI was calculated. Brachial blood pressure was recorded as an average of 3 supine measures taken over 10 min (DINAMAP-1846-SX, Critikon Corp., Tampa, Florida). Fasting venous blood was drawn for glucose, insulin, hemoglobin A1c (HbA1c), triglycerides, renal function, liver function, and free fatty acids. Insulin levels and HbA1c were checked in the patients with diabetes, but not in control subjects. Homeostasis model assessment of insulin resistance (HOMA-IR) was used to evaluate insulin resistance using the following equation: (fasting serum insulin [μU/l] × fasting plasma glucose [mmol·l^−1^])/22.5 (20).

### Cardiac CT

#### Coronary CT

An optional scan of coronary computed tomographic angiography (CCTA) was offered to patients with diabetes to exclude obstructive coronary artery disease (>50% of luminal stenosis) and for assessment of epicardial fat volumes. CCTA was performed with a GE VCT 64 slice scanner (GE Healthcare, Little Chalfont, United Kingdom) and a Snapshot Pulse protocol with prospective electrocardiographic triggering. Participants received beta-blockade (intravenous metoprolol) and sublingual glyceryl trinitrate prior to the scan to achieve a heart rate of <65 beats/min. During the CCTA acquisition, 70 ml of iodinated contrast (Niopam 370, Bracco, High Wycombe, United Kingdom) was injected at a rate of 6 ml/s followed by a 50-ml saline flush. The scan covered a region from 1 to 2 cm above the left main coronary artery to 1 to 2 cm below the myocardial apex in a single breath hold.

#### Epicardial fat volume quantification

CT images were reconstructed using medium-soft kernel (standard) with slice thickness of 0.625 mm and then transferred to a dedicated workstation for image processing (TeraRecon Aquarius iNtuition version 4.4.11, TeraRecon Inc., San Mateo, California). The adipose tissue volume was quantified using contrast-enhanced CT images. The layer of the epicardium was manually traced, and a 3-dimensional image was constructed using a semiautomated method. The volume was then calculated by a blinded operator (S.T.) and defined as the tissue with attenuation of −190 to −30 HU.

### Cardiac MR

All LV imaging was performed on a 3.0-T MR system (Siemens, Erlangen, Germany). Images for LV volumes and diastolic function were acquired using a steady-state free precession sequence and analyzed using cmr42 (Circle Cardiovascular Imaging Inc., Calgary, Alberta, Canada). To determine midventricular systolic circumferential strain and diastolic strain rate, myocardial tagging was performed 21, 22. Tagged images were analyzed using Cardiac Image Modeller software (CimTag2D version 7, Auckland Medical Research, Auckland, New Zealand). Semiautomated analysis was performed by aligning a grid to the myocardial tagging planes at end-diastole. A more detailed description of the MRI methods and MR acquisition parameters is included in the Online Appendix.

### ^31^P-magnetic resonance spectroscopy

^31^P-MRS was performed to obtain the rest PCr/ATP from a voxel placed in the midventricular septum, with the subjects lying prone with their heart over the center of the ^31^P heart/liver coil in the isocenter of the magnet. ^31^P-MRS post-processing analysis was performed using in-house software within Matlab version R2012a (Mathworks, Natick, Massachusetts). A more detailed description of the cardiac ^31^P-MRS acquisition parameters is included in the Online Appendix.

### Cardiac and liver ^1^H-MRS

Myocardial ^1^H-MRS was obtained from the midinterventricular septum. Liver triglyceride content was measured using ^1^H-MRS, avoiding vascular and biliary structures. Spectroscopic acquisitions were performed using electrocardiographic trigger. Water-suppressed spectra were acquired to measure myocardial and liver triglyceride content, and spectra without water suppression were acquired and used as an internal standard. Spectra were analyzed using Matlab and the AMARES algorithm in the Java-based Magnetic Resonance User Interface. Myocardial and liver triglyceride contents were calculated as a percentage relative to water: (signal amplitude of lipid/signal amplitude of water) × 100. A more detailed description of the ^1^H-MRS acquisition parameters is included in the Online Appendix.

### Liver MRI

The liver multiparametric MR protocol has been previously described (16). MR scans were performed using a 3-T scanner (Tim Trio, Siemens). Transverse abdominal T_1_ and T_2_* MR maps were acquired for the estimation of extracellular fluid and liver iron, respectively. Patients fasted overnight prior to their MRI scans.

### cT_1_ and fibroinflammatory liver disease

T_1_ relaxation time increases with increases in extracellular fluid, such as in fibrosis and inflammation. However, the presence of iron, which can be accurately measured from T_2_* maps, has an opposing effect on the T_1_. An algorithm has been created that allows for the bias introduced by elevated iron to be removed from the T_1_ measurements, yielding the cT_1_.

LiverMultiScan (Perspectum Diagnostics, Oxford, United Kingdom) is a software product specifically developed to measure cT_1_ from T_1_ and T_2_* maps. For this study, the LiverMultiScan was used to analyze anonymized images by investigators blinded to the clinical data (C.K., M.P.). cT_1_ was measured in a single, operator-defined region of interest away from vascular and biliary structures.

### Statistical analysis

All statistical analysis was performed with commercially available software packages (SPSS Statistics version 20, IBM, Armonk, New York). All data were checked for normality using the Kolmogorov-Smirnov test and presented as mean ± SD. Comparisons between the 3 groups were performed by 1-way analysis of variance with post hoc Bonferroni corrections. Bivariate correlations were performed using the Pearson or Spearman method as appropriate. The Student *t* test was used for comparison of normally distributed datasets where data were obtained for only 2 T2D groups. Significance was defined as p < 0.05.

## Results

### Participant characteristics

Demographic, clinical, and biochemical data are shown in Table 1. A total of 27 Ob-T2D patients (14 males, age 56 ± 8 years, BMI 33 ± 3 kg/m^2^, mean diabetes duration 6.1 ± 4.7 years, mean HbA1c 7.7 ± 1.4%), 15 Ln-T2D patients (9 males, age 56 ± 9 years, BMI 23 ± 2 kg/m^2^, mean diabetes duration 6.6 ± 6.5 years, mean HbA1c 7.4 ± 0.9%), and 12 healthy volunteers (8 males, age 50 ± 10 years, BMI 23 ± 2 kg/m^2^) were recruited. Participants in all groups were of similar age and sex, and there were no significant differences in blood pressure, diabetes duration, diabetes treatment, or metabolic profile between the 2 diabetes groups (Table 1). Systolic blood pressure was statistically higher in participants with T2D compared with control subjects, although it remained within normal limits. A total of 77% of the patients with diabetes were on statin therapy, and patients with diabetes therefore had lower low-density lipoprotein cholesterol levels compared with control subjects.

### Cardiac geometry and function

Cardiac MR results for LV volumes and function are summarized in Table 2. LV volumes and ejection fraction were similar between the Ln- and Ob-T2D groups and control subjects. However, although LV ejection fractions were not significantly different across the groups, more subtle functional changes with impairment in peak circumferential systolic strain and diastolic strain rates were evident in Ob-T2D compared with control subjects (p = 0.001 and p = 0.006, respectively) and also compared with Ln-T2D (p = 0.015 and p = 0.026, respectively). As we have previously shown, diabetes was associated with LV concentric hypertrophy (19), characterized by increased LV mass to volume ratio and increased LV mass, in both diabetes groups compared with control subjects.

### Cardiac metabolic phenotype

Cardiac ^1^H- and ^31^P-MRS results for myocardial triglyceride and energetics are summarized in Table 2. Diabetes was associated with cardiac steatosis even in the absence of obesity (Ln-T2D vs. control subjects; p = 0.01), and there was no significant difference in myocardial triglyceride levels between the Ob- and Ln-T2D groups. PCr/ATP was significantly reduced in both T2D groups compared with control subjects (Ob-T2D vs. control subjects; p = 0.002; Ln-T2D vs. control subjects; p = 0.043). There was no significant difference in myocardial PCr/ATP ratio between the Ob- and Ln-T2D groups (p = 0.92). There were no significant correlations between the myocardial PCr/ATP ratio and the markers of ectopic and visceral adiposity, such as hepatic triglyceride content (r = −0.17; p = 0.36), or with epicardial fat volume (r = −0.23; p = 0.27).

### Epicardial fat

**Figure 2:**
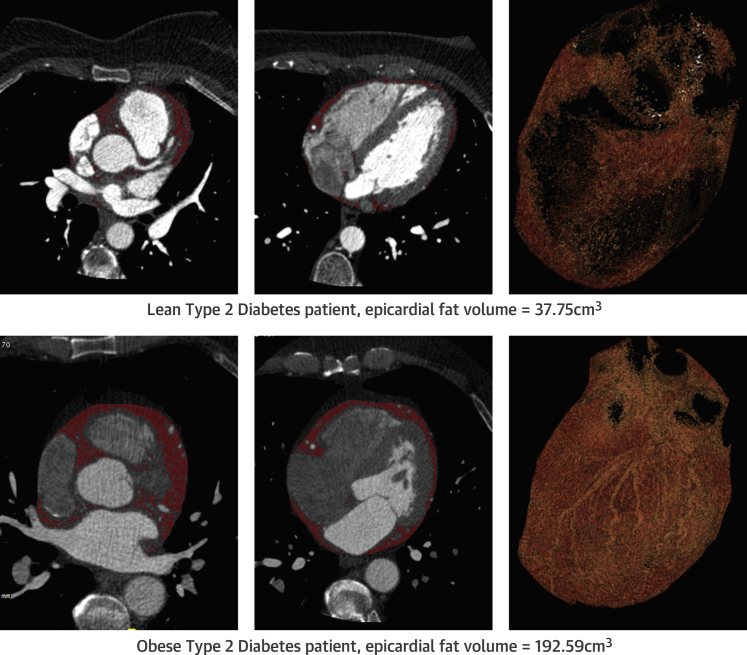
Representative Examples of CT Epicardial Fat Volumes in a Lean and an Obese Patient With T2D **(Top)** Lean patient with T2D with epicardial fat volume 37.75 cm^3^. **(Bottom)** Obese patient with T2D with epicardial fat volume 192.59 cm^3^. Abbreviations as in Figure 1.

Epicardial fat volume assessment was carried out in 33 patients (79% of the study patients) who have opted for CCTA. The Ob-T2D group had higher epicardial fat volumes compared with the Ln-T2D group (96 ± 40 cm^3^ vs. 71 ± 21 cm^3^; p = 0.04). Figure 2 shows representative images of epicardial fat volume in an LN- and an Ob-T2D patient.

### Hepatic steatosis, iron content, fibrosis, and inflammation

Liver enzymes and multiparametric liver MRI results for hepatic steatosis, fibrosis, and hemosiderosis are summarized in Table 3. Similar to cardiac steatosis, diabetes, even in the absence of obesity, was associated with hepatic steatosis (hepatic triglyceride content in Ln-T2D vs. control subjects; p = 0.044); however, hepatic steatosis was most marked in the Ob-T2D group: approximately 2-fold higher than in the Ln-T2D group (Ob-T2D vs. Ln-T2D; p = 0.012) and approximately 4-fold higher than in control subjects (Ob-T2D vs. control subjects; p = 0.005). Iron levels were normal across the groups.

**Figure 3:**
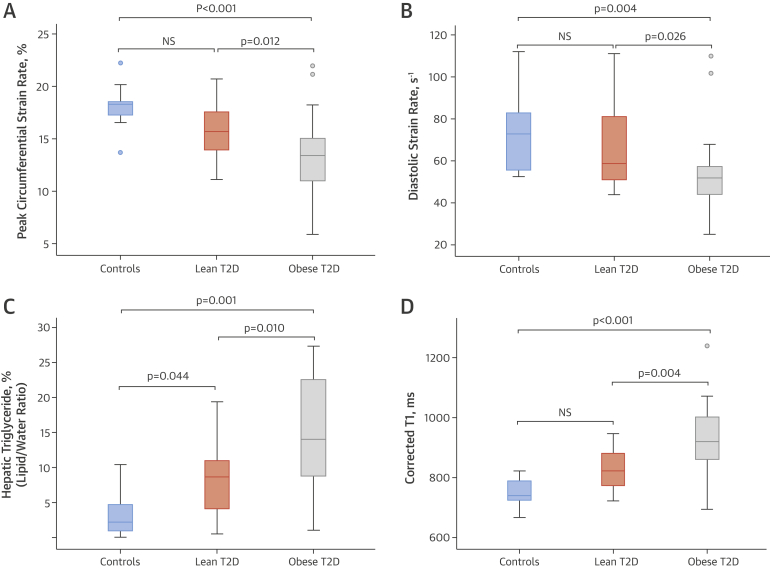
Differences in Cardiac Function, Hepatic Steatosis, and Hepatic cT_1_ Among the Study Cohorts **(A)** Peak circumferential systolic strain; **(B)** diastolic strain rate; **(C)** hepatic triglyceride content (%); and **(D)** hepatic corrected T_1_ map (ms). The **dots** indicate values outside the interquartile range. Abbreviations as in Figure 1.

Mean cT_1_ was highest in the Ob-T2D group, where the highest levels of hepatic triglyceride content were detected. The numeric differences in mean cT_1_ between Ln-T2D and control subjects did not reach statistical significance (p = 0.245), whereas cT_1_ in the Ob-T2D group was significantly increased compared with the Ln-T2D group (p = 0.004) and control subjects (p < 0.001), indicating significant fibroinflammatory liver disease in this group. Figure 3 represents differences in cardiac function, hepatic steatosis, and hepatic cT_1_ across the study cohorts. There was a positive correlation between the hepatic cT_1_ and hepatic triglyceride content (r = 0.71; p < 0.001). Importantly, despite the presence of hepatic steatosis and fibroinflammatory changes in the Ob-T2D group, there was no significant difference in liver enzymes compared with control subjects, and there was no association between liver cT_1_ and liver enzymes. Alanine aminotransferase levels were only minimally elevated (>45 to <80 IU/l) in 5 Ob-T2D patients and were normal in all other patients. The Central Illustration shows representative liver ^1^H-MR spectra, and a liver T_1_ map in a volunteer and in a lean and an obese patient with T2D.

### Relationship of insulin resistance, ectopic fat accumulation, and cardiac function

Insulin resistance, measured by HOMA-IR, was significantly higher in the Ob-T2D compared with the Ln-T2D group (p = 0.03). When investigating all T2D subjects, there was a positive correlation between the HOMA-IR and epicardial fat volumes (r = 0.47; p = 0.029), hepatic triglyceride (r = 0.39; p = 0.046), and hepatic cT_1_ (r = 0.58; p = 0.001), and there was a negative correlation between HOMA-IR and peak circumferential systolic strain (r = −0.52; p = 0.003). Furthermore, peak circumferential systolic strain also correlated negatively with the hepatic triglyceride (r = −0.49; p = 0.001) and epicardial fat volumes (r = −0.53; p = 0.004). Similarly, diastolic strain rate correlated negatively with hepatic triglyceride (r = −0.54; p < 0.001) and epicardial fat volumes (r = −0.59; p = 0.001). Myocardial triglyceride did not correlate with epicardial fat volumes (r = 0.36; p = 0.103) or with hepatic triglyceride (r = 0.23; p = 0.168).

## Discussion

This study demonstrates for the first time that diabetes, even in the absence of obesity, is associated with significant cardiac structural and metabolic abnormalities, whereas significant functional changes, such as reductions in peak systolic strain and diastolic strain rates, are only evident in obese patients with diabetes. Furthermore, we show that those patients with diabetes who are also obese have higher epicardial fat volumes, significant NAFLD, and higher insulin resistance. Importantly, we demonstrate here that the degree of hepatic and epicardial fat accumulation is associated with cardiac contractile dysfunction in diabetes. We confirm the findings of previous studies showing the association between epicardial fat deposition and insulin resistance 23, 24; moreover, we also demonstrate that there is an association between fibroinflammatory liver disease and insulin resistance in patients with diabetes. The correlation of systolic strain and diastolic strain rates with hepatic and epicardial fat and insulin resistance suggests a link between these in patients with diabetes. However, the causality of these relationships will need to be investigated in future studies.

Finally, as is widely known, the spectrum of NAFLD ranges from fatty liver alone to nonalcoholic steatohepatitis (25). We show here that diabetes, even in the absence of obesity, is associated with hepatic steatosis at the mild end of the liver disease spectrum, but not with significant fibroinflammatory liver disease. Importantly, using multiparametric liver imaging, we show that significant NAFLD and nonalcoholic steatohepatitis are present in asymptomatic patients with T2D with minor or no alanine aminotransferase elevation. This technique promises to answer a pressing need for a reliable, quick, and noninvasive screening, staging, and monitoring tool for diabetic liver disease.

### Ectopic and visceral fat, insulin resistance, and the heart

Our results suggest that Ln-T2D patients are likely to have less pronounced insulin resistance, lower levels of epicardial and hepatic fat accumulation, and better cardiac function than Ob-T2D patients. It is now widely accepted that adipose tissue is a dominant regulator of lipid and glucose metabolism (26). Multiple studies support the concept that insulin resistance is prompted, and sustained by, dysregulated fat tissue 27, 28, 29. In addition, insulin resistance and ectopic adiposity are associated with an even greater cardiovascular risk 30, 31, and obese subjects with T2D are at high risk of developing ectopic adiposity (32). There are many molecular mechanisms that may contribute to the association between insulin resistance and nonischemic cardiomyopathy (9). These include metabolic inefficiency (33), impaired vascular function (34), inflammation, and mitogenic actions of insulin on myocardium leading to changes of left ventricular geometry (35).

Epicardial adipose tissue has dichotomous functional characteristics, both adverse and protective, interacting locally with the coronary arteries and the myocardium through paracrine and vasocrine pathways. Under physiological conditions, epicardial fat supplies heat to the myocardium and exerts a protective effect on the coronary arteries 23, 36. Its pathological increase, and the coexistence of other metabolic and hemodynamic abnormalities, turn it into an adverse lipotoxic, prothrombotic, and proinflammatory organ (31).

In our study, the dissociation of myocardial steatosis from epicardial and liver fat is in keeping with a previous study in patients without diabetes and supports the hypothesis that myocardial lipid accumulation may represent a separate entity that is influenced by factors beyond visceral adiposity (37). Rijzewijk et al. (38) and McGavock et al. (39) previously demonstrated myocardial steatosis in patients with T2D. Furthermore, McGavock et al. (39) performed ^1^H-MRS in a large cohort of patients with T2D and were the first to show that hepatic triglyceride was not predictive of myocardial triglyceride (38). Thus, elevated levels of intracellular triglyceride in hepatocytes do not necessarily reflect elevated triglyceride levels in cardiac myocytes in T2D, which we confirm here.

### Ectopic fat and the liver

Our results suggest that Ln-T2D patients are more likely to have simple steatosis and Ob-T2D patients are more likely to have steatohepatitis. Our study is the first to date to noninvasively assess the severity of liver damage using a multiparametric MRI protocol in Ln- and Ob-T2D patients and to determine the effect of fibroinflammatory liver disease on the cardiac phenotype. We have demonstrated that asymptomatic Ob-T2D patients have significantly higher liver cT_1_ compared with Ln-T2D patients and healthy volunteers. This would indicate a greater burden of fibroinflammatory liver disease in this group of patients who should be prioritized for NAFLD screening in clinical practice. Importantly, these differences were present on imaging but not on alanine aminotransferase levels, suggesting that alanine aminotransferase alone is not a sensitive screening test for the presence of NAFLD in these patients. It has previously been shown that liver cT_1_ is associated with fibrosis (16) and also that it can differentiate simple steatosis from steatohepatitis 40, 41, 42.

NAFLD is defined as excessive fat accumulation in the liver (>5.6%) (43); it is among the leading causes of death in T2D (44) and is linked to hepatic insulin resistance (45). Despite this strong association and the emergence of NAFLD as a novel cardiovascular risk factor, only a few studies have addressed the presence of myocardial structural and functional changes in patients with NAFLD. Specifically, NAFLD, diagnosed either by ultrasonography or by liver biopsy, was shown to be associated with a higher prevalence of reduced coronary flow reserve (46), coronary calcification (47), impairment in diastolic function (48), concentric LV remodeling, and reduced longitudinal shortening (49).

### Study limitations

This study is limited by a relatively small sample size. Of 42 patients with T2D, 9 patients (21%) did not consent to have CCTA performed, and it is possible that occult coronary artery disease could be present in this minority of patients. CCTA was not performed in normal volunteers to avoid unnecessary radiation exposure. Significant coronary artery disease was deemed to be unlikely in this normal cohort, and epicardial fat volumes were therefore only assessed and compared in Ob- and Ln-T2D patients.

For assessment of liver disease, we have not used liver biopsy, the current gold standard, because it is invasive and limited by sampling and observer-dependent variability (50). Instead, we used a recently established, noninvasive, multiparametric scanning method, which has demonstrated a high diagnostic accuracy for the assessment of liver fibrosis, steatosis, and hemosiderosis (16).

Although the differences in mean peak systolic strain and diastolic strain rates in Ln-T2D compared with control subjects did not reach statistical significance, this may be due to the relatively small sample size. Larger studies of Ln-T2D patients are needed to confirm this. Although the release of adipokines including adiponectin and leptin has been considered among the important actions of adipocytes, we did not assess circulating levels of adiponectin or leptin.

There is evidence of a role for the sympathetic nervous system in the relationship between insulin and hypertension in obese patients with hypertension (51). We did not demonstrate any significant difference in resting heart rates or the systolic blood pressure between the 2 diabetes groups to suggest an enhanced adrenergic drive in the obese group, but we did not assess circulating catecholamine levels.

Finally, the observational nature of our findings precludes inferences of causality. Additional research is necessary to further delineate the relationship between ectopic and visceral adiposity with potential systemic effects such as insulin resistance and their role in the development of cardiac dysfunction in patients with T2D.

## Conclusions

Ob-T2D patients show a greater propensity than Ln-T2D patients for ectopic and visceral fat deposition that is associated with cardiac contractile dysfunction and fibroinflammatory liver disease.

Perspectives **COMPETENCY IN MEDICAL KNOWLEDGE:** Diabetes is associated with abnormalities of cardiac structure, energetics, and steatosis irrespective of BMI. Ob-T2D patients have a greater propensity for ectopic and visceral fat deposition, cardiac dysfunction, fibroinflammatory liver disease, and insulin resistance.

**TRANSLATIONAL OUTLOOK:** Further studies are needed to delineate the mechanistic relationships between ectopic and visceral adiposity, insulin resistance, and cardiac dysfunction in patients with T2D.

## Figures and Tables

**Central Illustration:**
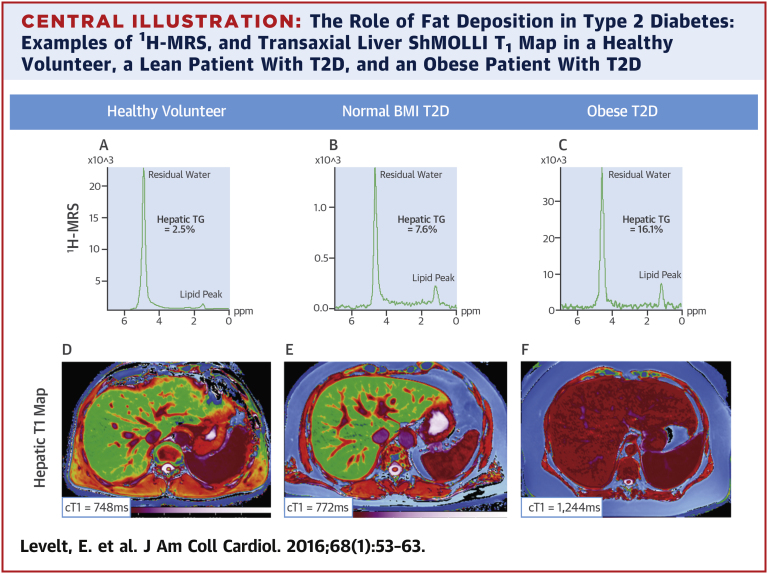
The Role of Fat Deposition in Type 2 Diabetes: Examples of ^1^H-MRS, and Transaxial Liver ShMOLLI T_1_ Map in a Healthy Volunteer, a Lean Patient With T2D, and an Obese Patient With T2D **(A)** Proton magnetic resonance spectroscopy (^1^H-MRS) of healthy volunteer with hepatic triglyceride (TG) 2.5%. **(B)**^1^H-MRS of lean patient with type 2 diabetes (T2D) with hepatic TG 7.6%. **(C)** Obese patient with T2D with hepatic TG 16.1%. **(D)** Healthy volunteer with liver Shortened Modified Look-Locker Inversion recovery (ShMOLLI) T_1_ map with corrected T_1_ (cT_1_) 748 ms. **(E)** Lean patient with T2D with liver ShMOLLI T_1_ map with cT_1_ 772 MS. **(F)** Obese patient with T2D with liver ShMOLLI T_1_ map with cT_1_ 1244 ms. BMI = body mass index.

**Table 1:** Clinical and Biochemical Characteristics

	Normal Control Subjects (n = 12)	Lean T2D Patients (n = 15)	Obese T2D Patients (n = 27)	p Value
Age, yrs	50 ± 10	56 ± 9	56 ± 8	0.163
BMI, kg/m^2^	23 ± 3	23 ± 2	33 ± 3∗	<0.001
Male	58	60	40	0.35
Diabetes duration, yrs	—	6.1 ± 4.7	6.6 ± 6.5	0.78
Heart rate, beats/min	66 ± 10	65 ± 7	69 ± 7	0.34
Systolic blood pressure, mm Hg	118 ± 14	131 ± 7†	130 ± 9†	0.002
Diastolic blood pressure, mm Hg	70 ± 8	76 ± 7	76 ± 7	0.05
Plasma fasting glucose, mmol/l	5.0 ± 0.5	8.1 ± 3.0†	9.5 ± 3.3†	0.001
Glycated hemoglobin, %	—	7.4 ± 0.9	7.7 ± 1.4	0.22
Hematocrit, %	43 ± 3	42 ± 3	43 ± 3	0.81
Insulin, pmol/l	—	107 ± 142	218 ± 255	0.03
HOMA-IR, %	—	1.26 ± 0.70	5.45 ± 5.6	0.03
Plasma triglycerides, mmol/l	0.92 ± 0.38	1.87 ± 1.81	1.75 ± 0.81	0.15
Plasma free fatty acids, mmol/l	0.59 ± 0.42	0.61 ± 0.20	0.67 ± 0.43	0.82
Total cholesterol, mmol/l	4.7 ± 1.0	3.8 ± 0.8	4.1 ± 1.0	0.10
HDL, mmol/l	1.55 ± 0.56	1.24 ± 0.29	1.20 ± 0.31†	0.03
LDL, mmol/l	2.93 ± 0.46	1.85 ± 0.59†	2.12 ± 0.82†	0.002
Medications				
Metformin	—	14 (93)	23 (85)	0.45
Sulfonylurea	—	4 (27)	12 (44)	0.27
Aspirin	—	2 (13)	7 (26)	0.35
Statin	—	8 (60)	19 (70)	0.51
ACE-I	—	7 (47)	10 (37)	0.56

Values are mean ± SD, %, or n (%).

ACE-I = angiotensin-converting enzyme inhibitors; BMI = body mass index; HDL = high-density lipoprotein; HOMA-IR = homeostasis model assessment of insulin resistance; LDL = low-density lipoprotein; T2D = type 2 diabetes.

∗ p < 0.05 versus lean T2D and control subjects with Bonferroni correction.

† p < 0.05 versus control subjects with Bonferroni correction.

**Table 2:** CMR and Cardiac MRS Findings

	Control Subjects (n = 12)	Lean T2D Patients (n = 15)	Obese T2D Patients (n = 27)	p Value
LV end-diastolic volume, ml	145 ± 40	124 ± 33	126 ± 25	0.15
LV ejection fraction, %	68 ± 5	73 ± 7	68 ± 8	0.11
LV mass, g	91 ± 30	123 ± 33∗	119 ± 28∗	0.01
LV mass index, g/m^2^	48 ± 11	66 ± 15∗	57 ± 10	0.001
LV mass to LV end-diastolic volume, g/ml	0.63 ± 0.13	0.95 ± 0.26∗	0.90 ± 0.20∗	<0.001
Peak systolic circumferential strain, negative (−), %	18.1 ± 2.1	16.5 ± 2.6	13.4 ± 3.6†	<0.001
Peak circumferential diastolic strain rate, s^−1^	74 ± 20	68 ± 19	56 ± 26†	0.006
Myocardial PCr/ATP ratio	2.08 ± 0.40	1.75 ± 0.29∗	1.64 ± 0.32∗	0.003
Myocardial triglyceride, % (lipid/water ratio)	0.48 ± 0.28	1.14 ± 0.66∗	1.22 ± 0.91∗	0.004

Values are mean ± SD.

CMR = cardiac magnetic resonance; LV = left ventricular; MRS = magnetic resonance spectroscopy; PCr/ATP = phosphocreatine to adenosine triphosphate concentration ratio; T2D = type 2 diabetes.

∗ p < 0.05 versus control subjects with Bonferroni correction.

† p < 0.05 versus lean patients with T2D and control subjects with Bonferroni correction.

**Table 3:** Liver Assessments

	Control Subjects	Lean T2D Patients (n = 15)	Obese T2D Patients (n = 27)	p Value
Liver enzymes				
Bilirubin, umol/l	12 ± 4	12 ± 6	11 ± 4	0.48
ALT, IU/l	22 ± 9	30 ± 22	36 ± 17	0.18
ALP, IU/l	145 ± 29	150 ± 50	163 ± 46	0.47
Albumin, g/l	44 ± 3	45 ± 2	46 ± 3	0.53
Multiparametric liver MRI				
cT_1_, ms	753 ± 45	821 ± 67	924 ± 116∗	<0.001
Hepatic triglyceride, % (lipid/water ratio)	3.8 ± 3.6	7.6 ± 4.6†	14.8 ± 8.4∗	<0.001
T_2_*, ms	20 ± 4	20 ± 4	18 ± 5	0.41
Liver iron, mg/g	1.3 ± 0.12	1.34 ± 0.13	1.33 ± 0.19	0.99

Values are mean ± SD.

ALP = alkaline phosphatase; ALT = alanine aminotransferase; cT_1_ = corrected T_1_; LIF = liver inflammation and fibrosis score; MRI = magnetic resonance imaging; other abbreviations as in Table 2.

∗ p < 0.05 versus lean patients with T2D and control subjects with Bonferroni correction.

† p < 0.05 versus control subjects with Bonferroni correction.
