# Supplementary material for: Ectopic and Visceral Fat Deposition in Lean and Obese Patients With Type 2 Diabetes
Source: J Am Coll Cardiol. 2016 Jul 5;68(1):53–63. doi: 10.1016/j.jacc.2016.03.597 (PMC4925621; doi:10.1016/j.jacc.2016.03.597)
Supplement: Online Appendix [file mmc1.docx]

**ONLINE APPENDIX**

**Supplemental Methods**

***Cardiac geometry and function***

Left ventricular (LV) volumes, mass and function were assessed using CMR imaging. Pilot images were acquired, followed by horizontal and vertical long axis cine images with the subjects in a supine position. A stack of steady-state, free precession (SSFP), short axis cine images were subsequently obtained using breath hold and cardiac gating.(1) The short axis images were obtained in a prospective manner, from the base to the apex. Each slice was 8 mm thick with a 3 mm gap with an echo time of 1.5 ms, repetition time 3 ms, and flip angle 50°. LV endocardium and epicardium of the short axis cine images were contoured using cmr42© (Circle Cardiovascular Imaging Inc, Canada). LV short-axis epicardial and endocardial borders were manually contoured at end-diastole and end-systole for determining end-diastolic volume (EDV), end-systolic volumes (ESV), and stroke volume (SV). Myocardial mass was also calculated by subtracting the endocardial volume from the epicardial volume. LV mass was calculated based on prior knowledge of myocardial density (1.05 g/cm^3^) and indexed to body surface area. LV mass to volume ratio was calculated by dividing the LV mass by the LV EDV.

*Strain imaging*

LV tagging was acquired to determine global midventricular systolic circumferential strain, as described previously.(2) A gradient echo-based high-resolution tagging pulse sequence was applied at the midventricular level of the LV. All data were prospectively acquired during breath-hold and ECG-triggering to capture 90% of the R-R interval. Sequence parameters were: field of view 360 mm, slice thickness 6 mm, TR 25 ms, TE 7.4 ms, flip angle 10°, and heart phase interval < 40 ms, giving 15–30 phases per cardiac cycle, depending on the heart rate. Tagged images were analyzed using Cardiac Image Modeller software (CimTag2D v7, Auckland Medical Research, Auckland, New Zealand). Semi-automated analysis was performed by aligning a grid to the myocardial tagging planes in end-diastole. End-systole was determined visually, and tags adjusted at each frame through the cardiac cycle to derive peak systolic circumferential strain for the midventricular slice, which was expressed as a percentage change from end-diastole.

***^31^P-MR Spectroscopy***

^31^P-magnetic resonance spectroscopy was acquired with a 3-dimensional ultra-short echo time chemical shift imaging (3D UTE-CSI) pulse sequence with acquisition-weighted and 10 averages at the center of k-space (3,4)An optimized radiofrequency pulse(4) centered between the γ- and α-ATP resonances was used to excitate all spectral peaks uniformly. Five nuclear Overhauser effect pulses (2.5 ms duration, 222.2 V_RMS_ and separated by 80.5 ms) were used to increase the signal-to-noise ratio. The acquisition matrix was 16×8×8 over a 240×240×200 mm^3^ field of view, placed with a central voxel in the midventricular septum and rotated to maximize coverage of the septal myocardium. Three 25-mm-thick saturation bands were used, 2 placed over chest wall muscle and 1 placed over liver proximal to the left ventricle. The total acquisition time was 9 minutes

All ^31^P-MRS spectra were processed and fitted within a custom Matlab (The Mathworks Inc., Natick, MA) program (5), which implemented the Advanced Method for Accurate, Robust, and Efficient Spectroscopic fitting (AMARES) algorithm (6). Before fitting the spectra were DC corrected. The spectra were fitted using prior knowledge(7) specifying 11 Lorentzian peaks (α,β,γ-ATP multiplet components, PCr, PDE, and 2×2,3-DPG) with fixed amplitude ratios and scalar couplings for the multiplets, and a fixed calibrated “begin time”.(5)

Spectral peak areas were corrected for the effects of nuclear Overhauser effect (NOE) enhancement using correction factors determined by prior experiment(4) : NOE correction factors were PCr 0.80, β-ATP 0.88, α-ATP 0.88, γ-ATP 0.79, DPG 0.70. Partial saturation was corrected using the flip angle computed from non-localized inversion recovery on a phenylphosphonic acid fiducial inside the coil housing, the protocol TR, and T_1_ values from literature:(8) PCr 3.8 s, γ-ATP 2.4 s, α-ATP 2.5 s, β-ATP 2.7 s, DPG 1.39 s, and PDE 1.1 s. The resulting peak areas of the 3 ATP signals were averaged and corrected for blood contamination by subtracting 11% of the sum of the two DPG peak areas.(9)

***Cardiac ^1^H-MRS for steatosis quantification***

Myocardial ^1^H-MR spectra were obtained from the mid-interventricular septum.(10) Spectroscopic acquisitions were performed using ECG triggering during a series of end-expiration breath-holds to minimize motion artefacts. Water-suppressed spectra were acquired to measure myocardial lipid content, and spectra without water suppression were acquired as an internal concentration reference. Spectra were analyzed in Matlab and using the AMARES algorithm implemented in the Java-based Magnetic Resonance User Interface (jMRUI version 4).(10) Myocardial lipid content was calculated as a percentage relative to water: (signal amplitude of lipid/signal amplitude of water)×100.

***Hepatic ^1^H-MRS for steatosis quantification***

*^1^H-MRS data acquisition*

Localizer images were obtained during end-expiratory breath-holds in the transverse, coronal and sagittal planes. After localization, a voxel was selected in the right lobe of the liver, avoiding blood vessels and the biliary tree. The voxel was typically placed half way between the porta-hepatis and the liver surface in order to avoid interference from the large fluid-filled structures of the porta-hepatis and the subcutaneous tissue and air which lie close to the liver periphery. A calibration pulse sequence was used to determine the optimum water suppression pulse scaling factor to better characterize the lipid peak at 1.3ppm. ECG-triggering was used to minimize noise from variations in arterial blood flow in the liver. A TR of 2 seconds allowed for complete relaxation of the lipid CH_2_ signal between successive RF pulses. Subjects had to hold their breath and lie still for 12 - 14 seconds. In total, 4 water suppressed spectra were acquired for lipid quantification and 1 non-water-suppressed spectrum for calibration. Spectroscopy parameters were (TE 10ms; mixing time 7ms; 1024 points acquired at a bandwidth of 2000Hz; scan frequency 1.3ppm for water-suppressed spectra and 4.7ppm for water unsuppressed spectra; TR 2s for water-suppressed data and 4s for water-unsuppressed data). Signals from different coil elements in each breath-hold were combined, and individual spectra phase- and frequency- corrected prior to summation.

*^1^H-MRS data analysis*

Spectra were analyzed using Matlab and the AMARES algorithm in Java-based Magnetic Resonance User Interface.(11) Hepatic lipid content was calculated as a percentage of the water content (signal amplitude of lipid / signal amplitude of water)×100.

***Hepatic T1 mapping***

*Image acquisition and analysis*

To minimize motion artefacts from respiration and the cardiac cycle, magnetic resonance (MR) data were acquired during expiratory breath-hold and with ECG-gating. This required subjects to repeatedly hold their breath and lie still for 12 - 14 seconds. A T1 relaxation time map was acquired using the Shortened Modified Look Locker Inversion recovery (shMOLLI) sequence (11) in a transverse plane through the mid-equatorial liver plane. A subject–dependent frequency adjustment was carried out during end-expiration. The ShMOLLI sequence samples the T1 recovery curve using single-shot steady state free precession (SSFP) acquisitions using the following parameters: TR 2.14ms, TE 1.07ms, flip angle of 35°, field-of-view optimized per patient, acquisition matrix 192x134-160, depending on patient, with GRAPPA acceleration of 2 with 24 reference lines, yielding a typical interpolated voxel size 0.9 x 0.9 x 8mm. Images were acquired 200ms after the ECG R-wave and the total time for each SSFP acquisition between 169 and 197ms, depending on the number of phase encoding steps.

*Region of interest placement*

A single region of interest (ROI) in the liver was selected for each patient. There were 4 considerations in the choice of the ROI. (a) As each acquisition generates an R^2^ map for the fit of signal intensity to the exponential recovery curve, the ROI was chosen in an area where R^2^ was ≥ 99. This was the case in all patients, (b) The ROI was placed approximately halfway between the porta hepatis and the liver surface in order to avoid interference from the fluid filled structures in the porta hepatis and subcutaneous tissue or air close to the liver surface, (c) The ROI was placed so as to avoid visible bile ducts and blood vessels, (d) The ROI was placed in an area that corresponded to good quality images in the T2* map in order to allow T1 and T2* quantification in the same ROI.

***Hepatic T2* mapping***

A multi-gradient-echo acquisition with RF spoiling was used to calculate a T2* map of the liver. The field-of-view was identical to the T1 mapping sequence, with a matrix size of 192x128- 160, depending on patient. A slice thickness of 3mm and 2x GRAPPA acceleration were used. Before acquisition, there was a delay of 200ms delay after the R-wave. The image was acquired in nine segments with a TR of 26.5ms and flip angle of 20°. Echo times were selected as far as possible such that the signals from fat and water were in phase (TE = 2.46, 7.38, 12.30, 17.22 and 22.14 ms). Fat-saturation and a double-inversion-recovery black blood preparation were used. The region of interest in the T2* maps was chosen along the principles described above for the T1 maps.

**Iron corrected T1**

The T1 measurements aim to quantify extracellular water resulting from fibrosis and inflammation. However, the presence of excess iron confounds this effect, leading to a T1 reduction. Elevated iron concentrations can be accurately quantified from T2* maps. In this work, we have used an algorithm to remove the confounding effect of iron from the T1measures, yielding the ‘iron-corrected T1’ (cT1; the T1 that would be measured using the shMOLLI sequence at a normal iron level; 1.3mg/g) (12)

**Supplemental References**

1. Hudsmith LE, Petersen SE, Tyler DJ et al. Determination of cardiac volumes and mass with FLASH and SSFP cine sequences at 1.5 vs. 3 Tesla: a validation study. J Magn Reson Imaging 2006;24:312-8.

2. Lawton JS, Cupps BP, Knutsen AK et al. Magnetic resonance imaging detects significant sex differences in human myocardial strain. Biomedical engineering online 2011;10:76.

3. Robson MD, Tyler DJ, Neubauer S. Ultrashort TE chemical shift imaging (UTE-CSI). Magnetic resonance in medicine : official journal of the Society of Magnetic Resonance in Medicine / Society of Magnetic Resonance in Medicine 2005;53:267-74.

4. Tyler DJ, Emmanuel Y, Cochlin LE et al. Reproducibility of 31P cardiac magnetic resonance spectroscopy at 3 T. NMR in biomedicine 2009;22:405-13.

5. Lucian A. B. Purvis, William T. Clarke, Luca Biasiolli, Matthew D. Robson, Rodgers CT. Linewidth constraints in Matlab AMARES using per-metabolite T2 and per-voxel ΔB0”. ISMRM 2014.

6. Vanhamme L, van den Boogaart A, S. VH. Improved method for accurate and efficient quantification of MRS data with use of prior knowledge. J Magn Reson 1997;129:35-43.

7. Zhang X, Heberlein K, Sarkar S, X. H. A multiscale approach for analyzing in vivo spectroscopic imaging data. Magn Reson Med 2000;43:331-334.

8. Bottomley PA, R. O. Optimum flip-angles for exciting NMR with uncertain T1 values. Magn Reson Med 1994;July:137-141.

9. Neubauer S, Krahe T, Schindler R et al. 31P magnetic resonance spectroscopy in dilated cardiomyopathy and coronary artery disease. Altered cardiac high-energy phosphate metabolism in heart failure. Circulation 1992;86:1820-1818.

10. Rial B, Robson MD, Neubauer S, Schneider JE. Rapid quantification of myocardial lipid content in humans using single breath-hold 1H MRS at 3 Tesla. Magnetic resonance in medicine : official journal of the Society of Magnetic Resonance in Medicine / Society of Magnetic Resonance in Medicine 2011;66:619-24.

11. Piechnik SK, Ferreira VM, Dall'Armellina E et al. Shortened Modified Look-Locker Inversion recovery (ShMOLLI) for clinical myocardial T1-mapping at 1.5 and 3 T within a 9 heartbeat breathhold. Journal of Cardiovascular Magnetic Resonance 2010;12:69-69.

12. Banerjee R, Pavlides M, Tunnicliffe EM et al. Multiparametric magnetic resonance for the non-invasive diagnosis of liver disease. Journal of Hepatology 2014;60:69-77.

13. Hoad CL, Palaniyappan N, Kaye P et al. A study of T₁ relaxation time as a measure of liver fibrosis and the influence of confounding histological factors. NMR Biomed 2015 Jun;28(6):706-14.
